# Optimization of the Heat-Drying Conditions of Drone Pupae by Response Surface Methodology (RSM)

**DOI:** 10.3390/foods12163062

**Published:** 2023-08-15

**Authors:** SeungHee Baek, Agapito Sheryl Mae, InSik Nam

**Affiliations:** 1 Research Center for Environmentally Friendly and Quality Livestock Production Technology, Hankyong National University, Anseong-si 17579, Gyeonggi-do, Republic of Korea; shbaek@hknu.ac.kr; 2 School of Animal Life Convergence Science, Hankyong National University, Anseong-si 17579, Gyeonggi-do, Republic of Korea; smvagapito@hknu.ac.kr

**Keywords:** edible insect, drone pupae, RSM, color, blanching, heat-drying, freeze-drying

## Abstract

Recent research has been conducted on various types of pre-processing methods for insects, including freeze-drying, microwave drying, hot air heat drying, and non-heat drying. This study aimed to identify the factors that have the greatest impact on heat drying conditions and establish the optimal heat drying conditions for drone pupae (*Apis melifera* L.) using response surface methodology (RSM) to minimize quality changes. Drone pupae were treated under various conditions, including blanching time (53–187 s) (X_1_), drying temperatures (41.6–58.4 °C) (X_2_), and drying time (266–434 min) (X_3_). The effect of these treatments on response variables, including the color parameter (WI, YI, BI, △E, and BD), AV, and TB of the dried drone pupae, was evaluated using a central composite design. The whole design consisted of 20 experimental points carried out in random order, which included eight factorial points, six center points, and six axial points. The optimal drying conditions for drone pupae were determined to be a blanching time of 58 s, a drying temperature of 56.7 °C, and a drying time of 298 min. The response variables were most affected by drying temperature and drying time and to a lesser extent by blanching time. The processed drone pupae using the optimized drying conditions resulted in the color parameters (WI, BI, YI, ΔE, and BD) being found to be 66.67, 21.33, 26.27, 31.27 and 0.13, respectively. And TB (log CFU/g) and AV (mg/g) values were found to be 3.12 and 4.33, respectively. The estimated and actual values for dried drone pupae showed no significant difference (*p* < 0.05). Comparing the physicochemical and microbiological properties of freeze-dried and optimal heat-dried drone pupae, the L and b value as well as PV were significantly lower in the heat-dried samples, while no significant difference was observed in the a value and AV (*p* < 0.05). Our study suggests that the model we developed can be applied to the large-scale production of drying conditions for use in the pharmaceutical and food industries.

## 1. Introduction

With the increased population and demand for higher protein sources, edible insects are gaining recognition as an alternative protein source [1]. The United Nations Food and Agriculture Organization (FAO) has proposed insect consumption as a viable substitute for meat [2]. It is projected that the edible insect market in the United States and Europe will reach approximately $3.3 billion by 2025, and in Asia, it is expected to grow to approximately $270 million by 2024. The global insect industry is anticipated to experience significant growth, with the market size expanding to tens of billions of dollars, particularly in sectors that utilize insects for applications such as new materials and pharmaceutical development [3].

Insects have advantages as a protein substitute food, but the challenge of overcoming consumer perception due to disgust for insects remains. To easily apply insects to food, insects can be processed into food ingredients such as insect powder or protein extract [4], but pre-processing is required for this. Pre-processing methods have a significant impact on the final product’s lipid oxidation, coloration, and microbial load. Insects undergo various reactions that cause color changes, including enzymatic polyphenol oxidation, complex formation between iron and polyphenols, and the formation of Maillard reaction products [5,6,7]. Traditional methods of pre-processing insects include roasting and sun-drying [8], and further research has been conducted recently on various types of pre-processing methods such as freeze-drying, microwave drying, hot-air heat drying, and non-heat drying. Browning is one of the main issues in several edible insect species [9]. Several enzymes, including phenol oxidase, lactase, tyrosine hydroxylase, decarboxylase, and peroxidase, play a role in the browning process. In insects, most phenolic compounds are produced from L-tyrosine in the shikimic acid pathway and are often modified by enzymes. Phenolic compounds can also react with proteins and amino acids non-enzymatically, resulting in the formation of dark compounds. Blanching is a widely used food pre-processing method for preparing insects in the food industry, and it is a common treatment aimed at deactivating enzymes, which can lead to undesirable changes in the food product. During the blanching process, samples are exposed to high temperatures ranging from 70 to 150 °C for a short period of time using hot water or steam [10,11]. Blanching is a method of immersing the material in boiling water, and when it is applied to sacrificing insects, hot water quickly penetrates into the insects, inhibiting denaturation during sacrifice, and it can also achieve enzyme inactivation and some sterilization effects, making it a sacrificial method with many advantages.

Bees are used as food in many countries around the world, such as Thailand and India [8,12,13,14,15], and in the case of bee larvae, there is evidence that it was used as a medicinal material in traditional East Asian medicine, such as ancient traditional Chinese therapies and Korean traditional medicine. Recently, research has been conducted on the drone pupae of the *Apis melifera* L. as a food ingredient in South Korea [16,17,18,19,20,21,22]. Drone pupae contain large amounts of major nutrients, amino acids, minerals, etc., and they have reported antioxidant, blood sugar enhancing, and hair loss improvement effects. However, after harvesting, the rich nutrients of drone pupae can be affected by lipid oxidation and microbial contamination depending on the storage method. After drone pupae are harvested, they should be stored in a freezer or freeze-dried, but most bee farms in Korea are small-scale farms with difficulties in facility investment. Therefore, it is necessary to develop a low-cost pre-processing method to reduce the production cost of drone pupae and to create high-value-added materials.

Response surface methodology (RSM) is defined as a statistical analysis method for the surface of a reaction where a dependent variable is affected by multiple independent variables with complex interactions [23]. These independent variables are factors that affect the response, and the dependent variable is a response variable that represents a reaction affected by the factors. The statistical approach involves setting up an appropriate experimental design based on independent variables (X_1_, X_2_, X_3_…) to obtain measured values of dependent variables (Y_1_, Y_2_, Y_3_…) and analyzing these variables to achieve the purpose of the study. This experimental design analysis is widely used in various aspects such as cost reduction, process development, product development, method development, and quality control. To optimize it, a one-factor-at-a-time method was traditionally applied in which all variables except one were fixed and only one variable was changed at a time to observe its effect. However, in cases where various independent variables interact with each other, such as in food research, a method of changing only one variable to study the interaction requires a lot of time and expense. Therefore, RSM, which can observe interactions between factors and save time and expenses, is widely used.

The purpose of this study was to investigate the factors that have the greatest influence on heat drying and to establish the optimal heat drying conditions for drone pupae using response surface methodology (RSM) to minimize quality changes such as discoloration and acidity according to these factors. To achieve this, we conducted experiments with blanching time, drying temperature, and drying time as independent variables for heat drying conditions, with color parameters, TB, and AV set as dependent variables. In addition, microbial and acidity changes were analyzed in drone pupae dried using optimized heat drying conditions and in those that were freeze-dried.

## 2. Materials and Methods

### 2.1. Materials

Ethanol, phenolphthalein solution, ethyl ether, and chloroform were obtained from Daejung Chemical & Metals Co., Ltd. (Seoul, South Korea). Potassium hydroxide solution (in ethanol) and acetic acid were obtained from Duksan Science (Seoul, South Korea). Potassium iodide and sodium thiosulfate pentahydrate were acquired from Sigma-Aldrich (St. Louis, MO, USA).

The drone pupae of *Apis mellifera* L. used in the experiment were raised in bee hives specifically at a beekeeping farm in Jeonbuk, South Korea in May 2022. The bee hives were removed after 20–23 days and immediately transferred to a freezer for storage before use in the experiment. The drone pupae in the bee hives were manually crushed, and they were separated and used. The drone pupae were blanched for 53–187 s in boiling water with a water:pupae ratio of 100:1 (*v:w*). After blanching, drone pupae were cooled in cold (4 °C) water to interrupt heat processes. And then, they were dried a laboratory oven for 266–434 min at 41.6–58.4 °C and used for experimental sample. For freeze-drying, the drone pupae blanched for 120 s in boiling water was first frozen at −70 °C for 48 h. The frozen sample was then freeze-dried using a freeze dryer (FD8512P, Ilshin-bio Co., Ltd., South Korea) at a temperature of −30 °C and pressure of 120 Pa for 48 h.

### 2.2. Color Parameters

#### 2.2.1. Color Indices

The color of each experimental sample was measured using a colorimeter (Color i7, X-rite, Grand Rapids, MI, USA) by taking five repeated measurements of the L* (lightness), a* (redness), and b* (yellowness) value. The standard white plate had an L value of 95.75, an a value of 0.15, and a b value of 2.82. The total color change (△E) was calculated using Equation (1). The L value difference (△L), a value difference (△a), and b value difference (△b) were analyzed using the initial value color difference. The whiteness (WI), yellowness (YI) and browning (BI) indices were calculated according to Tellez-Morales et al. [23] following Equations (2)–(4):

(1) $$△E=\sqrt{{\left(△L^{*}\right)}^{2}+{\left(△a^{*}\right)}^{2}+{\left(△b^{*}\right)}^{2}}$$

(2) $$\mathrm{WI}=\sqrt{\left(100-L^{*2}\right)+a^{*2}+b^{*2}}\;$$

(3) $$\mathrm{YI}=\frac{142.86\times b^{*2}\;}{L^{*2}}\;$$

(4) $$\mathrm{BI}=\frac{\left[100\times \left(X-0.31\right)\right]}{0.17}\;\;\mathrm{where}\;X=\frac{\left(a^{*}+1.75\times L^{*}\right)}{\left(5.645\times L^{*\;}+a^{*\;}-3.012\times b^{*}\right)}\;$$

#### 2.2.2. Browning Degree (BD)

BD was measured according to the method by Chang et al. [24]. Experimental samples were extracted by adding 5 g of the sample to 50 mL of 50% ethanol and extracting for 24 h at room temperature. The extracted solution was filtered through filter paper (Whatman No. 2), and the absorbance was measured at 420 nm using a UV spectrophotometer (Optizen 2120UV, Mecasys Co., Ltd., Daejeon, Republic of Korea).

### 2.3. Acid Value (AV)

AV was measured according to the method by Kimetal [18]. A five-gram sample was mixed with a 100 mL mixture of ethyl ether and ethanol (2:1, *v*/*v*), shaken, and then 100 μL of a 1% phenolphthalein solution was added as an indicator. The amount of 0.1 N potassium hydroxide solution required to titrate the sample until a light pink color was maintained for 30 s and used to calculate the acidity value (Equation (5)).

(5) $$\mathrm{AV}(\mathrm{mg}/g)=\frac{5.611\times \left(S-B\right)\times N\;}{Sample\;wt\;\left(g\right)}\;$$

where S = sample titration (mL); B = blank titration; N = normality of KOH–ethanol solution.

### 2.4. Peroxide Value (PV)

PV assays was carried out following the method by Kim et al. [18] with slight modifications. Briefly, 20 mL of acetic acid/chloroform (3:2 *v*/*v*) was used to dissolve a 1 g sample of oil, and then, a burette was used to add 1 mL of a saturated potassium iodide solution. Iodine was created after the reaction had occurred. Deionized water (50 mL) was added. Using a burette, iodine was titrated with 0.01 N sodium thiosulfate pentahydrate. The following Equation (6) was used to compute the peroxide value (expressed as milliequivalents of peroxide per kilogram of sample):

(6) $$\mathrm{PV}(\mathrm{meq}/\mathrm{kg})=\frac{\left(S-B\right)\times N\;}{Sample\;wt\;\left(g\right)}\;\times \;1000$$

where S = sample titration (uL); B = blank titration; N = normality of Na_2_S_2_O_3_

### 2.5. Microbial Analysis

Total aerobic bacteria and total coliform in the sample were determined using the methods described by Lee et al. [25].

#### 2.5.1. Total Aerobic Bacteria (TB)

Each 25 g sample was blended with 225 mL of sterile saline for 2 min using a stomacher (BagMixer^®^ 400, Interscience Inc., St. Nom, France). Then, a series of 10-fold serial dilutions was prepared using sterile saline, and each dilution (1 mL) was inoculated on Petrifilm aerobic count plates (Petrifilm™ 3M, St. Paul, MN, USA) that were incubated at 30 °C for 48 h.

#### 2.5.2. Total Coliform (TC)

To count total coliform, Petrifilm coliform count plates (Petrifilm™ 3M, St. Paul, MN, USA) were used, and the samples were incubated at 37 °C for 24 h. The identified colonies were counted and expressed as the log of the number of colony-forming units (CFUs)/g.

### 2.6. Experimental Design and Statistical Analysis

RSM was used to establish the optimization of the heat-drying conditions [26], and the optimization was performed using Minitab Statistical software Cloud-Based Version 20 (Minitab LLC., State College, PA, USA). A central composite design was employed to design the experimental data. The independent variables were set as blanching time (X_1_), drying temperature (X_2_), and drying time (X_3_) with a total 20 samples. Table 1 presents the values of α for the three independent variables, which were encoded into 5 levels, and the value of α was set to 1.68 [27]. Based on the results of preliminary experiments, the outline of experimental design with the coded and actual levels is presented in Table 2. The dependent variables (Y) were color parameters (WI, YI, BI, △E, and BD), TB and AV. These values were related to the coded variables by a quadratic polynomial using the equation below (7):

Y = β_0_ + β_1_X_1_ + β_2_X_2_ + β_3_X_3_ + β_11_X_1_^2^ + β_22_X_2_^2^ + β_33_X_3_^2^ + β_12_X_1_X_2_ + β_13_X_1_X_3_ + β_23_X_2_X_3_ (7)

The coefficients of the polynomial model were represented by β_0_ (constant term), β_1_, β_2_ and β_3_ (linear effects), β_11_, β_22_ and β_33_ (quadratic effects), and β_12_, β_13_ and β_23_ (interaction effects). Analysis of variance (ANOVA) was used to determine the significance of the regression coefficient, and the developed regression models were validated using the conducted statistical analysis. The adequacy of the model was evaluated by the coefficient of multiple determination (R^2^). The terms in the polynomial were considered statistically different when *p* < 0.05.

A *t*-test was used to determine the significance of differences between optimal heat-dried and freeze-dried drone pupae. Statistical analyses were performed using SPSS 22.0 for Windows (SPSS Inc., Chicago, IL, USA).

## 3. Results and Discussion

**Figure 1:**
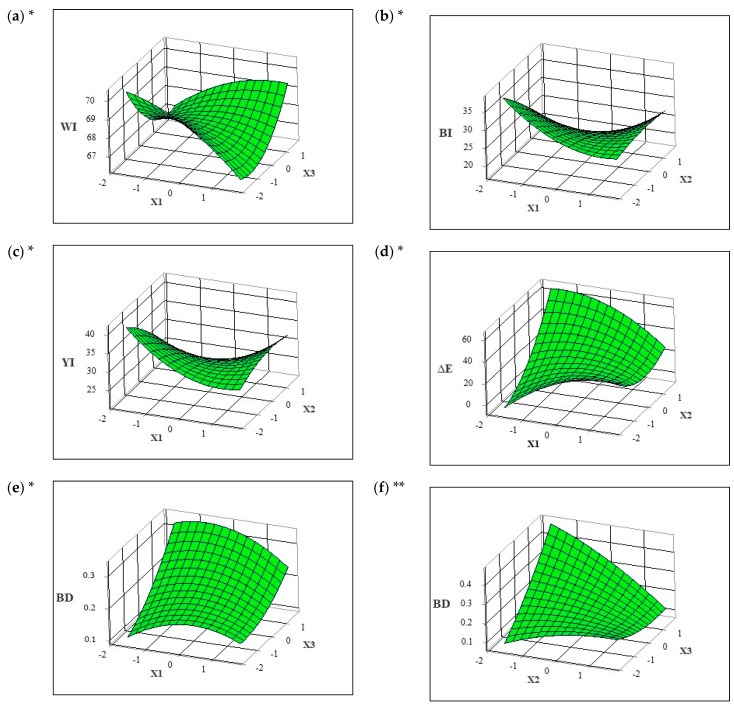
The three-dimensional response surface plots showing interactive effects of blanching time (X_1_), drying temperature (X_2_), and drying time (X_3_) on WI (**a**), BI (**b**), YI (**c**), △E (**d**), BD (**e**,**f**). Each net surface represents the response surface predicted with the quadratic model as a function of each variable and described by the equations given in Table 3. *: significant at *p* < 0.05. **: significant at *p* < 0.01.

For the sake of simplicity, some of the three-dimensional response surface plots are not depicted in Figure 1.

### 3.1. Model Fitting

The corresponding fitting of the explanatory models and the variation of the color parameters (WI, BI, YI, △E, and BD), TB, and AV were analyzed by the sum of squares of the sequential model. The estimated regression coefficients of the quadratic polynomial models for the response variables, along with the corresponding coefficients of determination (R^2^), are presented in Table 3. The coefficient of determination, R^2^, is the proportion of variation in the response attributed to the model rather than random error. It is recommended that an R^2^ value of at least 80% is necessary for a well-fitted model, and as the R^2^ value approaches unity, it indicates the empirical model’s suitability to the actual data. Conversely, a lower value of R^2^ indicates the unsuitability of the model to explain the relationship between variables [28]. Our results indicate that the R^2^ values for response variables were higher than 0.80. Specifically, the R^2^ values for WI, BI, YI, △E, BD, TB, and AV were found to be 0.8155, 0.9557, 0.9254, 0.9132, 0.9778, 0.9126, and 0.8693, respectively. These values suggest that the models accurately predicted the properties of these variables.

### 3.2. Color Parameters

The results in Table 3 indicate that the WI was statistically significant (*p* < 0.05) in the linear term of drying temperature (X_2_) and interaction term of blanching time and drying time (X_1_X_3_). The regression model fitted to the experimental results of the WI showed a correlation coefficient of R^2^ of 0.8155. The lack of fit (Table 4) was not significant, showing that these models are reliable predictors of responses. The negative coefficient of the linear term of drying temperature (X_2_) indicated that the WI decreased with an increase in drying temperature, which is to be expected by the dark color of the dried drone pupae. On the other hand, the positive coefficient of the interaction term (X_1_X_3_) of blanching time and drying time resulted in an increase in the WI. This is evident in Figure 1a; it was observed that the WI gradually increased with longer blanching and drying times. The results of this study are consistent with the findings of Cacchiarelli et al. [9], who reported higher WI values in larvae subjected to longer blanching times and higher blanching temperatures. For the BI (Table 3), it was observed that the significant (*p* < 0.001) effects were linear drying temperature (X_2_) and linear drying time (X_3_), interaction of blanching time and drying temperature (X_1_X_2_), quadratic blanching time (X_1_) and quadratic drying time (X_3_). Based on the sum of squares (Table 4), the importance of the independent variables on the BI could be ranked in the order of drying time (X_3_) and drying temperature (X_2_). Figure 1b describes the interaction between blanching time (X_1_) and drying temperature (X_2_). It was observed that the BI increased as the blanching time and drying temperature increased. Similarly, for the YI (Table 3), it was observed that the significant (*p* < 0.001) effects were linear drying temperature (X_2_) and linear drying time (X_3_), interaction of blanching time and drying temperature (X_1_X_2_), quadratic blanching time (X_1_) and quadratic drying time (X_3_). For blanching, although the linear effect of blanching time on the BI and YI was not significant, the coefficient of quadratic effects was significant. Based on the sum of squares (Table 4), the importance of the independent variables on the YI could be ranked in the order of drying time (X_3_) and drying temperature (X_2_).

In Figure 1d, samples dried under different drying conditions showed an increase in color change, with a significant increase in the △E value observed with increasing drying time. Based on the sum of squares, the importance of the independent variables could be ranked in the order of drying time (X_3_) and drying temperature (X_2_). Figure 1e,f show that the significant (*p* < 0.001; Table 3) interactions in the response variable for BD were all linear, quadratic, and interaction terms except for the interaction of blanching time and drying temperature (X_1_X_2_). The BD is defined as the degree of purity of the brown color and is one of the most commonly used indicators of browning in various food products [29]. These data are consistent with the WI, where higher drying temperatures trigger the Maillard reaction and caramelization, resulting in a darker hue and increased intensity of color in the drone pupae. After blanching, BD gradually increased with increasing drying time. This becomes more evident with the three-dimensional plot, where the linear blanching time and drying temperature term was significant (*p* < 0.001; Table 3) with a negative effect. On the other hand, BD gradually increases as the drying time increases. Therefore, the longer drying time causes the increase in BD, which is to be expected by the dark color of the dried drone pupae.

### 3.3. Total Aerobic Bacteria

The regression equation for expansion as TB is shown in Table 3. It was observed that the drying temperature and time had very significant positive linear effects (*p* < 0.001). Based on the sum of squares (Table 4), the importance of the independent variables could be ranked in the order of drying temperature (X_2_) and drying time (X_3_). It can be observed that there is an increasing trend in TB with drying temperature and time. During the drying of drone pupae, an increase in microbial counts is observed, which is expected due to the rich nutrients in them.

### 3.4. Acid Value

The regression equation for expansion as AV is shown in Table 3. It was observed that there is a decrease in AD (mg/kg) as the blanching time increases with the evaluation of drying temperature and drying time. However, when the changes in AD values were measured according to the drying time, it was observed that the AD value increased as the drying temperature increased. These findings are supported in Table 3, where significant effects (*p* < 0.05) were observed for the terms of linear blanching time (X_1_) (negative effect), drying temperature (X_2_) (positive effect), drying time (X_3_) (positive effect) and quadratic blanching time (X_1_) (negative effect). Therefore, it can be expected that the acid value of dried drone pupae would increase as a result of the drying treatment after blanching, since this treatment affects the oxidation of the product.

### 3.5. Optimal Drying Conditions

The optimal parameters for the development of drying conditions of drone pupae to improve pre-processing quality were found to be the minimum values of BI, YI, △E, BD, TB, and AV, as well as the maximum value of WI. The optimal drying conditions were determined to be a blanching time of 58 s, a drying temperature of 56.7 °C, and a drying time of 298 min. The predicted and actual values for the optimal drying conditions are given in Table 5. The difference of process parameters between the actual data and the statistically predicted data in the processed drone pupae with optimized drying conditions showed a minimum of 1.41% difference and a maximum of 29.38% difference. A 95% confidence level *t*-test (IBM SPSS Statistics 22) was used to compare the predicted and actual values for dried drone pupae in optimum drying condition circumstances, and no significant difference was detected.

### 3.6. Physicochemical and Microbiological Properties of Freeze Dried and Heat-Dried Drone Pupae

#### 3.6.1. Acid Value and Peroxide Value of Freeze-Fried and Heat-Dried Drone Pupae

To compare the AV and PV of freeze-dried drone pupae, measurements were taken of the heat-dried drone pupae optimized in this study using a commonly used preprocessing method for edible insects (Table 6). The AV did not show a significant difference between the two samples. However, the PV was higher in the heat-dried sample. The PV, as an indicator of the initial stage of oxidation spoilage, is represented by the amount of peroxide, which is a primary oxidation product formed during the initial oxidation process of lipid components. The PV of the freeze-dried sample was 5.74 ± 1.90 meq/kg, while that of the heat-dried sample was significantly higher at 10.36 ± 1.02 meq/kg. The processing of raw materials can significantly affect quality, nutritional composition, digestibility, and palatability in food manufacturing. This is particularly true for animal-based raw materials such as edible insects, which require a slaughtering stage and subsequent microbiological and chemical stabilization by drying to reduce water activity [14]. Defatting is a common step in the use of edible insects to produce high-protein products, which is the primary purpose for using them. Lipids can dilute protein concentration, hinder manufacturing technology, and affect palatability, as well as the productive yields and lipid composition of animals in the case of feed. The three main processes involved in processing insect larvae, which are slaughter, drying, and defatting, can significantly affect lipid oxidation in fats and meals. Blanching and freezing are the most popular methods for slaughtering insects. After testing different killing methods, blanching was preferred by Larouche et al. [30] for all quality parameters of black soldier fly (*Hermetia illucens*) larvae meals, including low lipid oxidation. Thermal methods, pro-oxidant conditions, and the degradation of minor antioxidant compounds due to processing can contribute to lipid oxidation. Both thermal methods (such as oven or microwave) and non-thermal methods (such as freeze-drying) have been tested for drying insect larvae, but conclusive results regarding the impact of drying methods on lipid oxidation have not been reached, which is likely due to dependence on the slaughtering method used [31,32]. In the present study, there was no significant difference in acid value between freeze-dried and heat-dried drone pupae.

#### 3.6.2. Color Indices of Freeze-Fried and Heat-Dried Drone Pupae

The results of the investigation into the color of freeze-dried and heat-dried drone pupae showed significant lowering of L and b values (*p* < 0.05) in the heat-dried drone pupae, which is consistent with a study conducted by Brishti et al. [33]. They suggested that the formation of brown pigments, such as melanoidins, through the Maillard reaction under heated conditions during oven drying may be the reason for the observed result. This finding is supported by the lowest amount of lysine content in oven dried, as lysine is actively involved in the Maillard reaction, and lower lysine content confirms the occurrence of the Maillard reaction. Color is an essential quality parameter associated with product or raw material quality attributes such as freshness, sensory and nutritional properties, and the presence of visual and non-visual defects. Additionally, color can indirectly control the Maillard reaction [34]. In the present study, it was observed that the color of the drone pupae became darker brown depending on the blanching time and drying conditions. The results of this study are similar to those found in other research. Cacchiarelli et al. [9] reported that insects that have undergone blanching treatment experience a decrease in water-holding capacity. They reported that when color parameters were measured in different pH solutions after boiling treatments (60 °C for 5 min and 90 °C for 1 min), WI and BI decreased more slowly than in the untreated control, indicating a browning effect. They also reported that the main effect on color differences (ΔE), which was calculated as the difference in color between time 0 and subsequent times, was due to the blanching treatment, which was followed by the effect of pH. Furthermore, these findings are consistent with the report of Saucier et al. [35] of a significant effect of blanching on black soldier fly larvae. They reported that pre-treatment of larvae with puncturing, blanching, and scalding in boiling water resulted in reduced drying times, which was presumably due to the impact on the wax-coated cuticle that shields the larvae from desiccation. Khatun et al. [36] investigated the color changes of freeze-dried, oven-dried, and blanched house crickets, and it was found that freeze-drying resulted in significantly higher levels of lightness than blanching and a lower browning index for cricket species. Another study observed the color of four insect species treated with the blanching method during low-temperature storage and reported that while the change in the L value was not significant over the storage period, there was a tendency for the a and b values to decrease due to the destruction of pigments and the formation of browning reaction products [37]. Kim et al. [38] conducted a study to measure the color of cricket powder that was freeze-dried and then oven-dried at various storage temperatures. The study found that at 25 °C, the L value increased slightly, but it decreased at 35 °C and 40 °C, with a trend of decreasing as the storage temperature increased over a long period of 6 months. Additionally, the a and b values decreased at all storage temperatures with a significantly greater reduction observed at higher temperatures compared to lower temperatures. Several studies have investigated the impact of freezing and blanching methods on the protein and lipid quality, as well as the color stability, of black soldier fly larvae. Leni et al. [39] reported that blanching, a method of killing insects, inhibits the browning reaction and other enzymatic changes that occur during slow killing by freezing. This leads to an increase in the extractability of proteins in aqueous solutions, prevents essential amino acid loss, and improves enzymatic digestibility. Caligiani et al. [40] reported that prepupae killed by freezing showed a significant reduction in acylglycerols during storage and the release of free fatty acids, which was likely due to the activation of lipases. In contrast, prepupae killed by blanching have a stable lipid fraction consisting mainly of triacylglycerols. Thus, the killing method has a significant impact on black soldier fly oil composition and potential applications. Larouche et al. [30] conducted a study to optimize larval killing methods and evaluate their impact on the nutritional and microbiological quality of black soldier fly larvae. Ten different methods were tested, resulting in varying the coloration of the freeze-dried and granulated larvae. Asphyxiation and cold-killing methods produced similar colors, while mechanical disruption and heating methods resulted in significantly different colors. Desiccation resulted in larvae with significantly higher color intensity than blanched or frozen larvae. High-pressure processing resulted in a product with significantly higher lightness and color intensity than grinding. Asphyxiation resulted in larvae with higher lightness and hue angle, resulting in a color closer to yellow compared to heat methods.

#### 3.6.3. Microbial Analysis of Freeze-Fried and Heat-Dried Drone Pupae

The total aerobic bacteria count was significantly higher in heat-dried drone pupae (2.99 Log CFUs/g) compared to freeze-dried (1.99 Log CFUs/g). Regarding total coliform, no colonies were detected after any treatment. In mulberry silkworm powder, the total microorganism count was 6.54 log CFUs/g. It was reported that frozen silkworms (*Bombyx Mori*), bamboo caterpillars (*Omphisa Fuscidentalis*), and field crickets (*Gryllus Bimaculatus*) had total aerobic counts of 7.9–8.3 log CFUs/g, yeast and mold counts of 5.5–6.2 log CFUs/g, and Enterobacteriaceae counts of 4.7–6.5 log CFUs/g [31,41]. Insects bred in contact with soil or sawdust are prone to contamination with various microorganisms, resulting in generally high microbial counts in insects. However, since the drone pupae grow in beehives and consume honey and royal jelly, their internal microbial count is lower compared to other edible insects.

## 4. Conclusions

To optimize the drying conditions of drone pupae, the response surface methodology (RSM) was used, and optimization was performed using Minitab software Cloud-Based Version 20. Our results showed that the R^2^ values for these response variables were higher than 0.80, indicating that the regression models were suitable to explain the behavior. The optimal drying conditions were determined to be a blanching time of 58 s, a drying temperature of 56.7 °C, and a drying time of 298 min. The difference in process parameters between the actual data and the statistically predicted data in the processed drone pupae with optimized drying conditions showed almost identical results, indicating that the pre-treatment conditions for edible insects using RSM are a suitable method. When comparing the physicochemical and microbiological characteristics of drone pupae processed by freeze-drying and drone pupae processed by optimized heat treatment, our results showed that the a value in color and acidity were not significantly different between the two groups. Our study suggests that the model we obtained can be applied to the large-scale production of drying conditions for further use in the pharmacy/food industries. Optimized conditions may be used in research involving test foods for the starting procedure of insect pre-processing for analysis of the samples. RSM is an effective tool for accounting for surface factors for an outcome and was used here to save labor and cost.

## Figures and Tables

**Table 1:** Experimental range and levels of the independent variables.

Independent Variable	Xi	Level				
		−1.68	−1	0	1	1.68
Blanching Time (s)	X_1_	53	80	120	160	182
Drying Temperature (°C)	X_2_	41.6	45	50	55	58.4
Drying Time (min)	X_3_	266	300	350	400	434

**Table 2:** Central composite experimental design for drying treatment with coded and actual levels of the independent variable.

Run	Point Type	Coded Variables			Actual Variables		
		X_1_	X_2_	X_3_	X_1_	X_2_	X_3_
1	Factorial	−1	−1	−1	80	45	300
2	Factorial	1	−1	−1	160	55	300
3	Factorial	−1	1	−1	80	45	300
4	Factorial	1	1	−1	160	55	300
5	Factorial	−1	−1	1	80	45	400
6	Factorial	1	−1	1	160	55	400
7	Factorial	−1	1	1	80	45	400
8	Factorial	1	1	1	160	55	400
9	Axial	−1.68	0	0	58	50	350
10	Axial	1.68	0	0	182	50	350
11	Axial	0	−1.68	0	120	41.6	350
12	Axial	0	1.68	0	120	58.4	350
13	Axial	0	0	−1.68	120	50	266
14	Axial	0	0	1.68	120	50	434
15	Central	0	0	0	120	50	350
16	Central	0	0	0	120	50	350
17	Central	0	0	0	120	50	350
18	Central	0	0	0	120	50	350
19	Central	0	0	0	120	50	350
20	Central	0	0	0	120	50	350

**Table 3:** Polynomial regression equations for color parameters (WI, BI, YI, △E, and BD), TB, and AV by independent variables.

Response Surface Model		R^2^	*p* Value
WI	=67.842 − 0.305 X_2_ − 0.399 X_3_^2^ + 0.521 X_1_X_3_	0.8155	0.010
BI	=24.187 − 2.792 X_2_ − 3.338 X_3_ + 1.456 X_1_^2^ − 1.132 X_3_^2^ + 1.798 X_1_X_2_	0.9557	<0.001
YI	=28.316 − 2.447 X_2_ − 3.250 X_3_ + 1.623 X_1_^2^ − 1.363 X_3_^2^ + 1.898 X_1_X_2_	0.9254	<0.001
△E	=27.62 + 8.94 X_2_ + 12.52 X_3_ − 4.22 X_1_^2^ + 4.71 X_2_^2^ + 7.02 X_3_^2^ − 6.74 X_1_X_2_	0.9132	<0.001
BD	=0.21796 − 0.00782 X_1_ − 0.03660 X_2_ + 0.04627 X_3_ − 0.01752 X_1_^2^ − 0.01155 X_2_^2^ + 0.00985 X_3_^2^− 0.01046 X_1_X_3_ − 0.04085 X_2_X_3_	0.9778	<0.001
TB	=3.345 + 0.741 X_2_ + 0.563 X_3_ + 0.3209 X_1_^2^	0.9126	<0.001
AV	=0.12702 − 0.01017 X_1_ + 0.00643 X_2_ + 0.01034 X_3_ − 0.00629 X_1_^2^	0.8693	<0.001

**Table 4:** Analysis of variance results and determination coefficients for RSM models.

Source	WI			BI			YI			△E		
	Sum of Squares	F Value	*p* Value	Sum of Squares	F Value	*p* Value	Sum of Squares	F Value	*p* Value	Sum of Squares	F Value	*p* Value
Model	10.8781	4.91	0.010	351.424	23.99	<0.001	342.858	13.79	<0.001	5280.84	11.69	<0.001
Linear	2.2419	3.04	0.080	264.158	54.09	<0.001	228.516	27.57	0.001	3230.05	21.46	<0.001
X_1_	0.9111	3.70	0.083	5.780	3.55	0.089	2.723	0.99	0.344	0.47	0.01	0.925
X_2_	1.2715	5.17	0.046	106.331	65.32	<0.001	81.692	29.57	<0.001	1090.89	21.74	0.001
X_3_	0.0593	0.24	0.634	152.047	93.40	<0.001	144.101	52.17	<0.001	2138.69	42.63	<0.001
Crossed	4.4175	5.98	0.013	31.442	6.44	0.011	36.601	4.42	0.032	1349.23	8.96	0.003
X_1_X_2_	1.1317	4.60	0.058	25.853	15.88	0.003	28.82	10.43	0.009	256.05	5.1	0.047
X_1_X_3_	2.1728	8.83	0.014	2.301	1.41	0.262	5.294	1.92	0.196	318.38	6.35	0.030
X_2_X_3_	1.1129	4.52	0.059	3.288	2.02	0.186	2.487	0.90	0.365	708.71	14.13	0.004
Quadratic	4.2187	5.71	0.015	55.824	11.43	0.001	77.741	9.38	0.003	701.56	4.66	0.028
X_1_^2^	0.7061	2.87	0.121	30.435	18.70	0.002	37.848	13.70	0.004	363.48	7.24	0.023
X_2_^2^	0.8109	3.30	0.100	1.478	0.91	0.363	5.724	2.07	0.181	135.5	2.7	0.131
X_3_^2^	2.2912	9.31	0.012	18.423	11.32	0.007	26.688	9.66	0.011	202.58	4.04	0.072
Lack-of-fit	2.0833	5.02	0.055	13.485	4.83	0.055	23.745	6.12	0.034	455.14	9.77	0.013
Pure error	0.3777			2.794			3.879			46.59		
Residual	2.4609			16.279			27.624			501.73		
Total	13.3391			367.703			370.482			5782.57		
**Source**	**BD**				**TB**				**AV**			
	**Sum of Squares**	**F Value**	***p* Value**		**Sum of Squares**		**F Value**	***p* Value**	**Sum of Squares**		**F Value**	***p* Value**
Model	0.07100	49.04	<0.001		14.9090		11.60	<0.001	0.00445		7.39	0.002
Linear	0.04832	100.14	<0.001		11.9674		27.92	<0.001	0.00344		17.12	<0.001
X_1_	0.00084	5.19	0.046		0.1504		1.050	0.329	0.00141		21.09	<0.001
X_2_	0.01828	113.65	<0.001		7.4924		52.45	<0.001	0.00057		8.44	0.016
X_3_	0.02921	181.58	<0.001		4.3246		30.27	<0.001	0.00146		21.82	<0.001
Crossed	0.01459	30.24	<0.001		0.3561		0.83	0.507	0.00034		1.69	0.232
X_1_X_2_	0.00037	2.30	0.160		0.0023		0.02	0.902	0.00014		2.14	0.174
X_1_X_3_	0.00088	5.44	0.042		0.1430		1.00	0.341	0.00005		0.71	0.418
X_2_X_3_	0.01335	82.97	<0.001		0.2107		1.48	0.252	0.00015		2.21	0.168
Quadratic	0.00808	16.75	<0.001		2.5856		6.03	0.013	0.00067		3.36	0.063
X_1_^2^	0.00441	27.42	<0.001		1.4796		10.36	0.009	0.00057		8.49	0.015
X_2_^2^	0.00192	11.92	0.006		0.5011		3.51	0.091	0.00014		2.02	0.185
X_3_^2^	0.00140	8.67	0.015		0.4673		3.27	0.101	0.00000		0.02	0.892
Lack-of-fit	0.001335	4.87	0.054		1.0166		2.47	0.172	0.000256		0.62	0.694
re error	0.000274				0.4119				0.000413			
Residual	0.001609				1.4286				0.000669			
Total	0.072607				16.3376				0.005116			

**Table 5:** Predicted and experimental values of the responses at optimum conditions.

Process Parameter	Target	Predicted Data	Actual Data	% Difference
WI	Maximize	69.502 ^a^	66.67 ± 0.56 ^a^	4.16 ± 0.84
BI	Minimize	22.898 ^a^	21.33 ± 0.43 ^a^	7.09 ± 2.04
YI	Minimize	25.909 ^a^	26.27 ± 0.48 ^a^	1.41 ± 1.79
△E	Minimize	25.55 ^a^	31.27 ± 9.79 ^a^	28.43 ± 15.46
BD	Minimize	0.135 ^a^	0.13 ± 0.01 ^a^	4.54 ± 3.59
TB (log CFU/g)	Minimize	4.193 ^a^	3.12 ± 0.12 ^a^	29.38 ± 3.61
AV (mg/g)	Minimize	4.114 ^a^	4.33 ± 0.18 ^a^	4.98 ± 4.19

Actual data and % difference indicate mean ± SD of triplicate measurements. Means within a row with same letters are not significantly different (*p* < 0.05).

**Table 6:** The comparison of the physicochemical and microbiological properties of the drone pupae treated with optimal heat drying and freeze drying.

Parameter	Freeze Dried	Heat Dried	*p* Value
L value	69.03 ± 0.49 ^a^	63.23 ±0.60 ^b^	0.008
a value	2.60 ± 0.22 ^a^	1.66 ±0.17 ^a^	0.093
b value	20.27 ± 0.82 ^a^	12.73 ±0.29 ^b^	0.003
AV (mg/g)	3.83 ± 0.06 ^a^	4.20 ± 0.26 ^a^	0.062
PV (meq/kg)	5.74 ± 1.90 ^b^	10.36 ± 1.02 ^a^	0.033
TB (log CFU/g)	1.99 ± 0.35 ^b^	2.99 ± 0.09 ^a^	0.032

All values are mean ± SD of triplicate measurements. Means within a row with different letters are significantly different (*p* < 0.05).

## Data Availability

The data are available from the corresponding author.
